# Global analysis of X-chromosome dosage compensation

**DOI:** 10.1186/jbiol30

**Published:** 2006-02-16

**Authors:** Vaijayanti Gupta, Michael Parisi, David Sturgill, Rachel Nuttall, Michael Doctolero, Olga K Dudko, James D Malley, P Scott Eastman, Brian Oliver

**Affiliations:** 1 Laboratory of Cellular and Developmental Biology, National Institute of Diabetes and Digestive and Kidney Diseases, National Institutes of Health, 50 South Drive, Bethesda, MD 20892, USA; 2 Incyte Genomics, Palo Alto, CA 94304, USA; 3 Mathematical and Statistical Computing Laboratory, Division of Computational Bioscience, Center for Information Technology, National Institutes of Health, Bethesda, MD 20982, USA; 4 Current address: Quantum Dot Corporation, Hayward, CA 94545, USA

## Abstract

**Background:**

*Drosophila melanogaster *females have two X chromosomes and two autosome sets (XX;AA), while males have a single X chromosome and two autosome sets (X;AA). *Drosophila *male somatic cells compensate for a single copy of the X chromosome by deploying male-specific-lethal (MSL) complexes that increase transcription from the X chromosome. Male germ cells lack MSL complexes, indicating that either germline X-chromosome dosage compensation is MSL-independent, or that germ cells do not carry out dosage compensation.

**Results:**

To investigate whether dosage compensation occurs in germ cells, we directly assayed X-chromosome transcripts using DNA microarrays and show equivalent expression in XX;AA and X;AA germline tissues. In X;AA germ cells, expression from the single X chromosome is about twice that of a single autosome. This mechanism ensures balanced X-chromosome expression between the sexes and, more importantly, it ensures balanced expression between the single X chromosome and the autosome set. Oddly, the inactivation of an X chromosome in mammalian females reduces the effective X-chromosome dose and means that females face the same X-chromosome transcript deficiency as males. Contrary to most current dosage-compensation models, we also show increased X-chromosome expression in X;AA and XX;AA somatic cells of *Caenorhabditis elegans *and mice.

**Conclusion:**

*Drosophila *germ cells compensate for X-chromosome dose. This occurs by equilibrating X-chromosome and autosome expression in X;AA cells. Increased expression of the X chromosome in X;AA individuals appears to be phylogenetically conserved.

## Background

In most organisms, copy number at any given locus has little effect on proper organismal function. Very few genes are deleterious if present in only one copy (haploinsufficiency) or are overtly deleterious in three copies. Having more or fewer copies (aneuploidy) of a large fraction of the genome is, however, invariably incompatible with viability. For example, over 10% of human oocytes are aneuploid, but with a few exceptions none of these aneuploid oocytes gives rise to viable offspring [1]. The most common aneuploid genotypes in a wide range of species involve the deletion or duplication of a chromosome or chromosome segment. Deletions are the most deleterious.

In *Drosophila melanogaster*, a systematic study of aneuploids with deletions of different segments of chromosomes indicates that having only a single copy of 1% of the genome reduces viability (and often fertility) and having only a single copy of 3% or more of the genome is lethal [2]. From current estimates of gene content in *Drosophila*, 3% represents about 500 genes [3]. Therefore, having only a single copy of 500 genes or more usually results in the collapse of a major part of the genetic network. That genetic networks do indeed collapse because of minor differences in the expression levels of a few connected nodes is evident from genetic interaction studies. In the female germline, for example, the dose of the gene *ovarian tumor *(*otu*) strongly modifies the sterility phenotype of flies heterozygous for *ovo*^*D *^[4]. (The gene *ovo *encodes a transcription factor that acts on *otu *[5]). Similarly, in the male germline, heterozygosity for *haywire *or *β-tubulin *mutations are tolerated, but heterozygosity for both results in failed spermatogenesis [6].

The sex chromosomes represent an extraordinary exception to the genetic imbalance rule. *Drosophila *males have one copy of the X chromosome per diploid set of autosomes (X;AA) and females have two (XX;AA) [7]. As the *Drosophila *X chromosome bears about 20% of the genome [3], *Drosophila *males vastly exceed the usual 3% single-copy threshold for viability. This is not due to an underrepresentation of dosage-sensitive genes on the X chromosome, as females are sensitive to X-chromosome deletions [2]. Therefore, males have a special mechanism(s) to compensate for X-chromosome dose (for reviews see [8,9]). An extensive set of autoradiographic experiments on the giant polytene chromosomes of the salivary gland showed that the X chromosome in X;AA flies is expressed at roughly twice the level as an X chromosome in XX;AA flies. Hypertranscription of the X chromosome in karyotypic males is dependent on a complex of at least five proteins and two non-coding RNAs. The genes encoding the proteins in the complex are referred to as the *male specific lethal (msl)* loci (*msl*). Males lacking any of the *msl *activities show reduced X-chromosome transcription and die as larvae. At the molecular level, these genes encode a histone-modifying MSL complex, which acetylates histone 4 on lysine 16 (H4 K16). The modification is thought to relieve the general repressive action of histones and result in increased transcription.

Interestingly, the MSLs do not function in the germline. The X chromosomes of male germ cells are not decorated with MSL complexes and are not hyperacetylated at H4 K16 [10]. Furthermore, neither the genes encoding the MSL complex nor the obligate somatic regulators of the MSLs are required for germline viability [11,12]. There is similar lack of evidence of dosage compensation in the germline of other organisms [13,14], leading to the hypothesis that germ cells are dosage-tolerant. Alternatively, dosage compensation in germ cells may be MSL-independent. Whether the germline X chromosome of *Drosophila*, or indeed of any organism, is dosage compensated is one of the major unresolved issues in the study of sex chromosomes. Our array results indicate that *Drosophila *germ cells do, in fact, dosage compensate.

Equally enigmatic are the dosage-compensation systems in *Caenorhabditis elegans *and mammals, which are based on reducing X-chromosome expression in XX;AA cells [15,16]. This is seen most clearly in mammals, where one of the X chromosomes in XX;AA females is inactivated. In *C. elegans*, both the X chromosomes in XX;AA hermaphrodites show reduced expression. In both cases, the dosage-compensation model equilibrates X-chromosome expression between the sexes but it also makes both sexes functionally aneuploid with respect to the autosomes. Both males and females (or hermaphrodites) become functionally X;AA. It has been suggested that this is counterintuitive, as within each diploid X;AA organism, gene expression from a single X chromosome should be equilibrated and balanced to the autosomes [17-20]. Therefore, it is more useful to think of X-chromosome dosage as a mechanism for equilibrating X chromosome and autosome expression, rather than as only a mechanism for equilibrating expression between the sexes. This predicts, for example, that in mammals both the single X chromosome of males and the single active X chromosome in females are hypertranscribed [17,18]. While there is an overwhelming literature supporting X-chromosome inactivation, there has been very little experimental evidence to support hypertranscription of the active X chromosome [21]. Our examination of array results in *C. elegans *and the mouse suggests that such X-chromosome hypertranscription does occur.

## Results

### Comparing XX;AA and X;AA expression ratios

**Figure 1:**
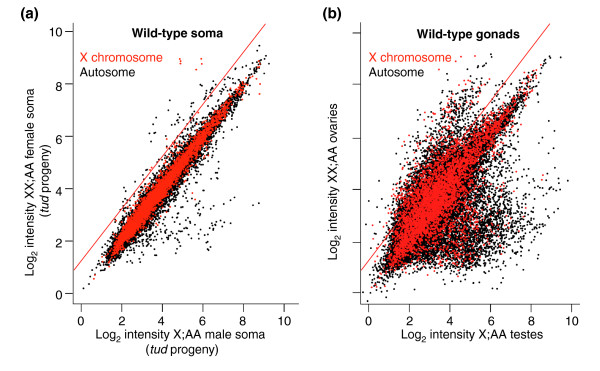
Scatterplots of hybridization intensities from wild-type female and male tissues. Hybridization intensities for XX;AA vs X;AA are plotted along the *y*-and *x*-axis respectively. Data points correspond to elements reporting autosomal genes (black) and X-chromosome genes (red). **(a) **Germlineless XX;AA female progeny of homozygous *tudor*^1 ^(*tud*^*1*)^) mothers (*tud*^+ ^being required for germ cell formation) compared with their germlineless X;AA male siblings; **(b) **XX;AA wild-type ovaries compared with X;AA wild-type testes from siblings. The expected twofold difference in gene expression in the absence of X-chromosome dosage compensation is shown as a red line.

Gene expression in XX;AA and X;AA tissues can be simply visualized by plotting the hybridization intensities of the two samples in a scatterplot (Figure 1). There is modest sex-biased expression in the soma, but extensive sex-biased expression in the gonads. In the absence of dosage compensation, we would expect to see XX tissue expression at twice the level of X tissues. There is no evidence of such a distribution of X-chromosome expression ratios in either tissue. The bulk of the XX versus X expression data points overlies the bulk of the AA versus AA expression data points in both tissues. This indicates that the single X chromosomes of X;AA soma and gonads are expressed at the same level as the two X chromosomes in XX;AA soma and gonads. This strongly suggests that somatic dosage compensation is widespread in adult tissues. More importantly, this provides the first strong evidence that X-chromosome dosage compensation occurs in the germline. It is, however, much more difficult to look for subtle effects of gene dose in the germline because of the dramatic and extensive sex-biased expression observed between ovaries and testes: this sex bias affects about 31% of the genome and results in a greater than tenfold difference in expression level for some genes.

**Figure 2:**
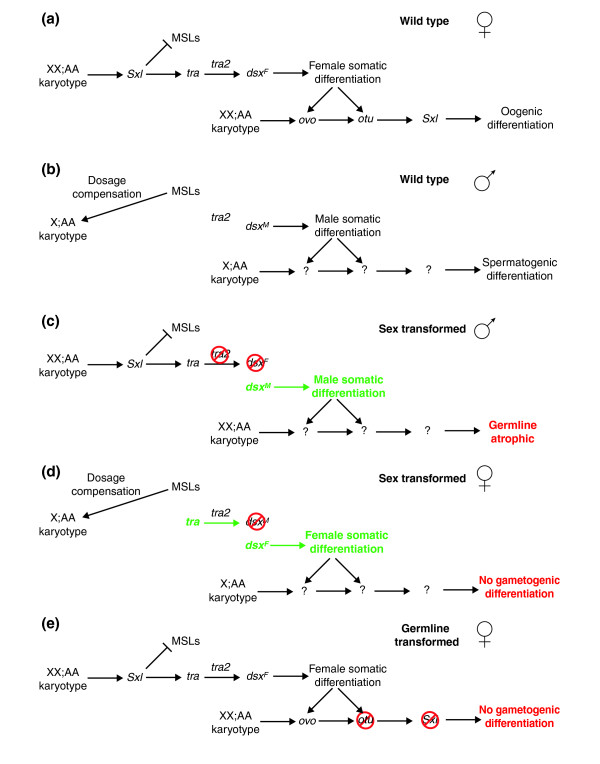
Sex-determination hierarchy. Sex-biased expression was controlled for by using mutations in sex-determination genes. Relevant aspects of **(a) **wild-type female, **(b) **wild-type males, **(c) **somatic transformation from female to male (sex transformed), **(d) **somatic transformation from male to female (sex transformed), and **(e) **germline transformation are outlined. (a) Sex determination occurs in early embryogenesis, before the activation of dosage compensation, which leads to higher levels of expression of transcription factors on the X chromosome in XX;AA than X;AA individuals. These transcription factors activate *Sex-lethal *(*Sxl*) in the soma. The Sxl protein regulates the alternative splicing of the *transformer *(*tra*) pre-mRNA such that Tra protein is produced only in females. Sxl also inhibits the formation of the MSL dosage compensation complex. Tra protein and non-sex-specifically expressed Transformer2 (Tra2) protein control the alternative splicing of the *doublesex *(*dsx*) pre-mRNA. The *dsx *mRNAs resulting from Tra- and Tra2-mediated splicing encode a female-specific Dsx^F ^transcription factor. Sex determination in the germline is poorly understood and controversial, but a female somatic environment and an independent reading of the XX;AA karyotype in germ cells increases expression of positively acting Ovo transcription factors and their direct target, *ovarian tumor *(*otu*). The *otu *locus is required for *Sxl *activity in the germline. Note that *Sxl *does not regulate the MSLs in the germline. The female sex-determination hierarchy results in oogenic differentiation. (b) In X;AA flies Sxl protein is not present. This permits the formation of the MSL dosage-compensation complex. The *tra *pre-mRNAs are spliced to a non-coding form in the absence of Sxl, and in the absence of Tra protein the *dsx *pre-mRNA is spliced into a default form encoding a male-specific Dsx^M ^transcription factor. The germ cells develop into sperm. (c) XX;AA flies are transformed from females into males using null mutations of *tra2 *and by using a *dsx *mutation encoding a pre-mRNA that is constitutively spliced into the male-specific form (*dsx*^*swe*^). Flies bearing *dsx*^*swe *^*in trans *to a deletion produce Dsx^M ^protein and no Dsx^F ^protein. Similarly, flies null for *tra2 *produce only Dsx^M^. To remove germline expression from the analysis of somatic X chromosome dosage compensation we took advantage of the fact that XX;AA flies transformed from females into males usually have no germline. These germline-atrophic (having few to no germ cells) XX;AA females transformed into males were compared with X;AA male carcasses (everything but the gonads) or with X;AA males with a genetically ablated germline due to the absence of maternal *tud*^+^. (d) X;AA flies are transformed from males into females by expressing female-specific *tra *cDNA transgenes. The activation of female rather than male sexual differentiation in the X;AA soma results in vast numbers of non-differentiated germ cells, presumably due to sexual incompatibility between the soma and germline. The sexual identity of these cells is ambiguous. (e) Mutations in *Sxl *(using allelic combinations effecting only in the germline isoforms of Sxl) or *otu *also result in vast numbers of non-differentiated germ cells. Positive (arrows) and negative (barred lines) genetic or molecular regulation are indicated. Loss-of-function (red) and gain-of-function (green) mutations and phenotypes are indicated.

**Figure 3:**
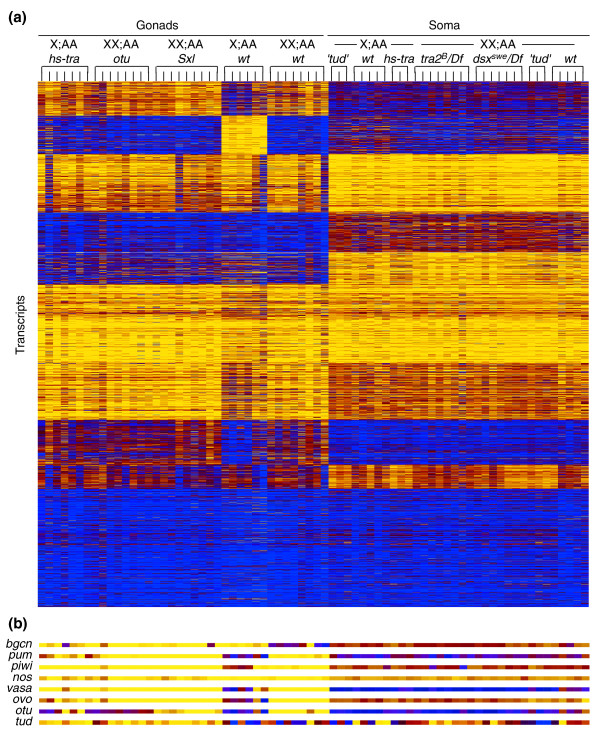
Germline-biased gene expression in transformed ovaries. **(a) **Heat diagram (yellow > red > blue) of hybridization intensities for all unique array element sequences (*N *= 13,267) from individual samples (columns) used in this study. Gonad samples (left) and somatic samples (right) are indicated with karyotype (XX;AA and X;AA) and abbreviated genotypes; wt, wild type (see Materials and methods for more details). Replicates are indicated (brackets). Germline expression is clearly evident in the gene-expression profiles of transformed ovaries. There are large blocks of elements showing high- or low-intensity hybridization to gonad probes and the opposite pattern when hybridized to samples from carcasses or from flies lacking germ cells but having somatic components of the gonads. **(b) **Selected genes with known functions in the germline. Array elements representing germline-marker genes (for example, *vasa *(*vas*), *pumilio *(*pum*), *tudor *(*tud*), *piwi *and *benign gonial cell neoplasia *(*bgcn*) [67,68]) show strong hybridization to labeled gonad mRNA samples and comparatively weaker hybridization to non-germline samples. Furthermore, at least some of the differences between the samples also support the proposed germline sex-determination pathway. For example, as predicted, both *ovo *and *otu *are germline-biased and overexpressed in XX;AA *Sxl *ovaries relative to X;AA *hs-tra *ovaries [40,69]. All these data validate the use of XX;AA and X;AA transformed germlines as matched tissues for the careful analysis of X-chromosome dosage compensation in the germline.

To avoid distortion due to sex-biased expression, we included sex-transformed tissues in our experiments (see Materials and methods). These tissues were produced by genetic manipulation of the sex-determination hierarchy (Figure 2, and see [7,22] for reviews). We use gonad samples to assay germline dosage compensation. Although both wild-type and transformed gonads are composed of germline and somatic tissues, germline cytoplasm accounts for the bulk of the mRNA [4,11,23]. We confirmed that the germline contributes the majority of the transcripts in these hybridizations, as we were unable to extract sufficient RNA from similar numbers of dissected empty gonads [24]. More importantly, we directly verified the composition of the sample gonads by looking for clusters of genes with germline-biased expression and for individual genes with known germline-specific or germline-biased expression patterns (Figure 3). These samples allow us to isolate the effect of X-chromosome dose from the confounding effects of sexual dimorphism.

**Figure 4:**
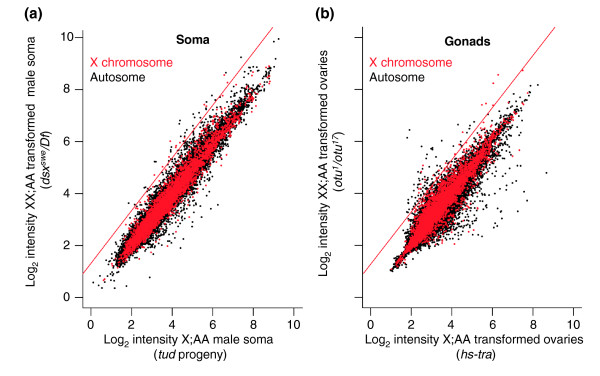
Scatterplots of hybridization intensities from transformed XX;AA and X;AA tissues. Data points correspond to elements reporting autosomal genes (black) and X-chromosome genes (red). **(a) **XX;AA females transformed into somatic males (*dsx*^*swe*^/*Df(3R)dsx*^*M*+*15*^) compared with germlineless X;AA male progeny of homozygous *tud*^*1*^mothers and **(b) **XX;AA ovaries from *ovo*^*1*^/*otu*^*17*^females compared with X;AA ovaries from *hs-tra*^*83*^/+ males transformed into females. The expected twofold difference in gene expression in the absence of X-chromosome dosage compensation is shown as a red line.

Scatter in the XX;AA versus X;AA data due to sex-biased expression can be very effectively reduced by sex transformation (Figure 4). When we compare expression of XX;AA and X;AA soma that were sexually matched (for example, females versus males transformed into females, and males versus females transformed into males), we observe very little high-magnitude differential expression. Even more striking is the similarity in the expression profiles of transformed ovaries bearing non-differentiating germ cells. The high-magnitude differences in expression observed between wild-type XX;AA ovaries and X;AA testes (Figure 1) are nearly abolished when we compared XX;AA and X;AA transformed ovaries (Figure 4). The implications from visual examination of the scatterplots are clear, as hybridization to array elements corresponding to X-chromosome and autosomal genes show expression ratios centered tightly on unity. We confirmed the veracity of this exploratory analysis using multiple statistical tools (see the section Transcriptional response to altered autosomal gene dose, below). This strongly supports the idea that X-chromosome dosage compensation occurs in the germline.

**Figure 5:**
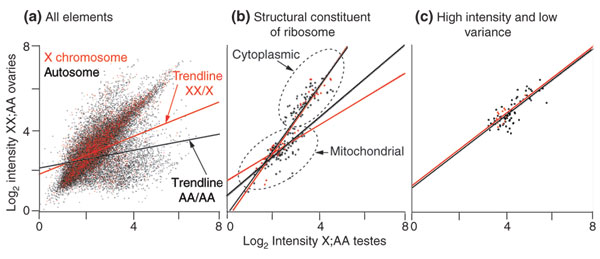
Subsets of gene-expression profiles from wild-type gonads. Scatterplots of hybridization intensities for XX;AA ovary (*y*-axis) versus X;AA testis (*x*-axis) samples to elements corresponding to **(a) **all genes, **(b) **genes encoding cytoplasmic or mitochondrial proteins, and **(c) ***de facto *housekeeping genes (showing high intensity and low variance across all experiments). Data points and regression lines (trendlines) correspond to elements reporting autosomal genes (black) and X-chromosome genes (red).

It is formally possible that X chromosomes are dosage compensated in XX;AA versus X;AA transformed germ cells but not in wild-type oogenic and spermatogenic germ cells. Although most of the data points lie along the diagonal in scatterplots of hybridization intensities comparing XX;AA ovaries and X;AA testes, regression lines for X chromosome and autosome expression profiles are differentially deflected (Figure 5). This is caused by the large number of genes showing male-biased expression on the autosomes and the lower density of genes with male-biased expression on the X chromosome [25]. There are, however, many genes with 'housekeeping' functions on both the X chromosome and the autosomes that are expected to be equally expressed in both oogenic and spermatogenic germ cells.

To determine how genes with housekeeping function respond to dose in the germline, we examined in more detail the expression levels of nuclear genes encoding cytoplasmic or mitochondrial ribosomal subunits [26]. The X chromosome and autosomal genes show strikingly similar distributions (*p *> 0.1 by regression analysis of slopes and intercepts), despite a twofold difference in X-chromosome dose (Figure 5b). We also identified a *de facto *set of housekeeping genes by investigating array elements showing reduced hybridization variance and high intensity hybridization across all our experiments (Figure 5c). In the absence of dosage compensation, we would expect a deficit of X chromosome genes in this subset, as they would show increased variance due to the dose difference. We found no deficit of X-chromosome encoded genes showing low-variance expression. Both these analyses indicate that at least some X-chromosome genes are dosage compensated in the wild-type germline.

### Transcriptional response to altered autosomal gene dose

To understand the precision of X-chromosome dosage compensation, we first need to understand the general response of the biological system to altered gene dose. What should we expect if there is no X-chromosome dosage compensation? A twofold difference in gene dose is unlikely to always result in a twofold difference in steady-state transcript levels, as many genes are regulated by elegant feedback mechanisms that would dampen the effect of gene dose. Similarly, we need to prove that the entire data-handling pipeline allows us to see gene-expression differences associated with altered gene dose.

To determine whether differences in genetic dose result in detectable differences in gene expression, we have directly measured gene-expression changes resulting from altered gene dose due to deficiency (deletion) and duplication of an autosomal segment of the genome. The duplication that we used overlaps the deletion region, but covers more genes. Flies heterozygous for the duplication *Dp(2:2)Cam3*/+ (*Dp*/+) have three copies of around 330 genes, while flies heterozygous for the deletion *Df(2L)J-H*/+ (*Df*/+) are hemizygous for a subset of around 70 of those duplicated genes [27]. By directly comparing gene expression in flies bearing these aberrations, we assayed the consequences for gene expression of 1-fold, 1.5-fold, and 3-fold differences in gene dose along chromosome arm 2L.

**Figure 6:**
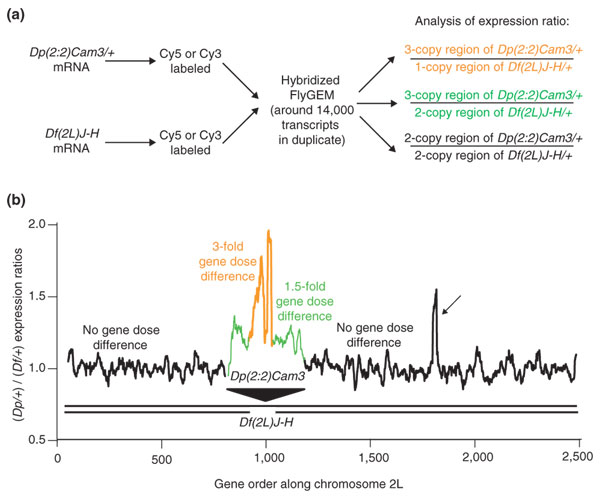
Gene-expression changes due to an altered gene dose on autosomes. **(a) **Experimental design to measure gene-expression changes for altered autosomal gene dose. Replicate mRNA samples from flies bearing a duplication, *Dp*(*2;2*)*Cam3*/+, or a deletion, *Df*(*2L*)*JH*/+, on the left arm of autosomal chromosome 2 (2L) were labeled with either Cy3 or Cy5 and hybridized to FlyGEM arrays. These were performed as dye-swap experiments to avoid any effect of dye bias. Following data extraction, changes in expression ratios as a result of differences in the dose of individual genes were determined. There are no differences in underlying gene dose outside the region of the aberrations, and there is a 1.5-fold difference when a gene is present on the duplication (*Dp*) or a 3-fold difference when the gene is present on the duplication and deleted in the deficiency (*Df*). **(b) **Average expression ratios from duplicate experiments comparing *Dp*/+ vs *Df*/+ adult male flies were plotted as a moving average against gene position along 2L. Gene-expression ratios for no gene dose difference (black), 1.5-fold (green) and 3-fold (orange) gene doses are indicated. Breaks in gene-expression ratios occur at the predicted cytological breakpoints of the aberrations. The aberrations are cartooned below the moving average plot, with the *Dp *(triangle) and *Df *(gap) indicated. In addition, the diploid regions of the genome where *Dp*/+ flies show greater expression than *Df*/+ flies are indicated (arrow).

We isolated mRNA from two independent preparations of females, males and ovaries and performed six hybridizations comparing *Dp*/+ and *Df*/+ samples directly against each other on microarrays (Figure 6). The effect of altered autosomal gene dose is obvious in the moving average plots of expression ratios against position along the chromosome arm (Figure 6). We observed distinct alterations of gene-expression ratios between *Dp*/+ and *Df*/+ flies within the cytologically defined aneuploid regions in males, females and ovaries. Moving from left to right along such a plot, the ratios are essentially 1.0 (indicating equivalent expression) in the region that is two-copy in both *Dp*/+ and *Df*/+ flies, until reaching the region that is three-copy in *Dp*/+ flies and two-copy in *Df*/+ flies. At this position there is a strong break in the moving average towards *Dp*-biased expression. A similar break in the moving average occurs at the transition to the region that is three-copy in *Dp*/+ flies and single-copy in *Df*/+ flies. Altered fold ratios between *Dp*/+ flies and *Df*/+ flies return to baseline as the proximal breakpoints of the aberrations are crossed.

The differences in gene-expression ratios corresponding to 1-, 1.5- and 3-fold dose changes on chromosome 2L are highly significant (*p <<*10^-4 ^by ANOVA followed by Tukey HSD, Hodges-Lehmann, and Kolmogorov-Smirnov tests; see Materials and methods). No other similar large blocks of genes elsewhere in the genome show significantly greater expression in *Dp*/+ flies than in *Df*/+ flies. There were, however, smaller two-copy regions that showed expression ratios mimicking the 1.5- to 3-fold gene dose effect or the inverse effect. These regions could be due to unrecognized aberrations or could be secondary transcriptional effects.

We also used these 1-, 1.5- and 3-fold dose-change data to tune our data handling. The on-spot background correction method gave the greatest discrimination power (see Materials and methods and Additional data file 1), and was used for all subsequent distribution analyses. This method maximizes our ability to detect the absence of dosage compensation.

The salient feature of the above analyses is that expression changes due to altered gene dose changes on autosomes are readily detectable. This provides us with a metric for the analysis of the similar X-chromosome gene-expression ratios when comparing XX;AA with X;AA tissues. We performed 48 hybridizations using 96 biological samples (see Materials and methods). Matched XX;AA and X;AA tissues were compared in direct hybridizations and were linked through the loop design to other XX;AA and X;AA samples hybridized on other arrays. This allowed for even greater numbers of comparisons. In all of our global comparisons of XX;AA with X;AA expression in the soma (72 comparisons), a 1.5-fold dose difference on an autosome gave a greater difference in gene-expression profiles than did a twofold difference in X-chromosome dose (*p *<< 10^-4^). The same was found in all of our global comparisons of XX;AA to X;AA expression in transformed gonads (119 comparisons). These data conclusively demonstrate that germ cells carry out dosage compensation.

### Mechanism of X-chromosome dosage compensation

Balancing X-chromosome expression between XX;AA and X;AA germ cells in *Drosophila *is likely to require either hypertranscription of the X in males (as occurs in the soma) or hypotranscription of the X chromosome in females compared with an autosomal reference. Determining which mechanism of dosage compensation operates in the germline requires measuring the absolute expression levels of the X chromosome genes relative to those of autosomal genes. Gene expression varies by orders of magnitude, but in a large representative population of genes on a chromosome arm we expect that those represented by two copies will have higher transcript abundance than those represented by a single copy.

**Figure 7:**
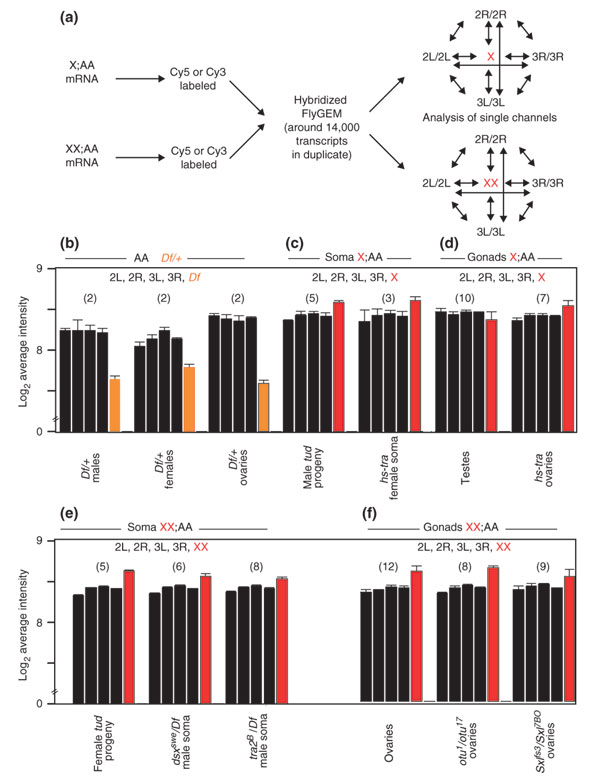
Increased X-chromosome expression in the X;AA soma and germline. **(a) **Experimental design. The mRNA samples from XX;AA or X;AA somatic or germline tissues were labeled with either Cy3 or Cy5 and hybridized to FlyGEM arrays. Hybridization intensities corresponding to X chromosomes (red) and autosomal genes (black) were analyzed within each X:AA or XX:AA sample. **(b-f) **Histograms of hybridization intensities. (b) Autosomal hybridization intensities from single-copy (orange) and two-copy (black) regions of *Df*/+ samples in whole adult males, whole adult females and ovaries. Bars represent autosomal arms, in the order shown across the top. (c-f) Average hybridization intensities from the single X chromosome (red) compared to two copies of each autosomal arms. (c) Germlineless X;AA male progeny of homozygous *tud*^*1*^mothers and carcasses from X;AA males transformed into somatic females with *hs-tra*. (d) X;AA gonads from wildtype males and X;AA *hs-tra *sex transformed flies. (e) Germlineless XX;AA female progeny of homozygous *tud*^*1*^mothers and XX;AA females transformed into males by *dsx*^*swe*^/*Df(3R)dsx*^*M*+*15*^and *tra2*^*B*^/*Df(2R)trix*. (f) XX;AA gonads from wild-type and *otu*^*17*^/*otu*^*1*^and *Sxl*^*7BO*^/*Sxl*^*fs3*^transformed ovaries. Error bars show standard deviation of the mean hybridization intensity from all replicates of each sample. Numbers of hybridization replicates are indicated in parentheses above each histogram panel.

As a rigorous feasibility test, we examined hybridization intensities in the *Df*/+ region of autosome 2L. In whole adult males, adult females and ovaries, average hybridization intensities were significantly lower in the 70 or so genes in the single-copy *Df *segment than in the two-copy segments elsewhere in the genome (Figure 7). X chromosomes are hypertranscribed in the soma relative to autosomes. As expected, transcripts from the single X chromosome in the soma hybridize to arrays as intensely as those from the two-copy autosomes. We observed the same trend in the X;AA germline. Transcripts from the X chromosome of X;AA transformed ovaries show as high an intensity of hybridization as those from the two-copy autosomes. In fact, the X chromosome appeared to be slightly overexpressed relative to autosomes in the X;AA transformed ovaries, in each of seven replicate experiments. Even the transcripts from the single X chromosome in X;AA testis showed higher intensity hybridization than the single-copy genes from any *Df+ *sample (*p *<< 10^-4^). These results indicate that X-chromosome dosage compensation in the *Drosophila *soma and germline is the result of higher per-copy X-chromosome transcript accumulation in X;AA cells. Interestingly, the X chromosome shows slight, but highly significant, overexpression in all XX;AA samples. This may be due to inherent hypertranscription of X-chromosome genes in both males and females.

### Escape from dosage compensation

**Figure 8:**
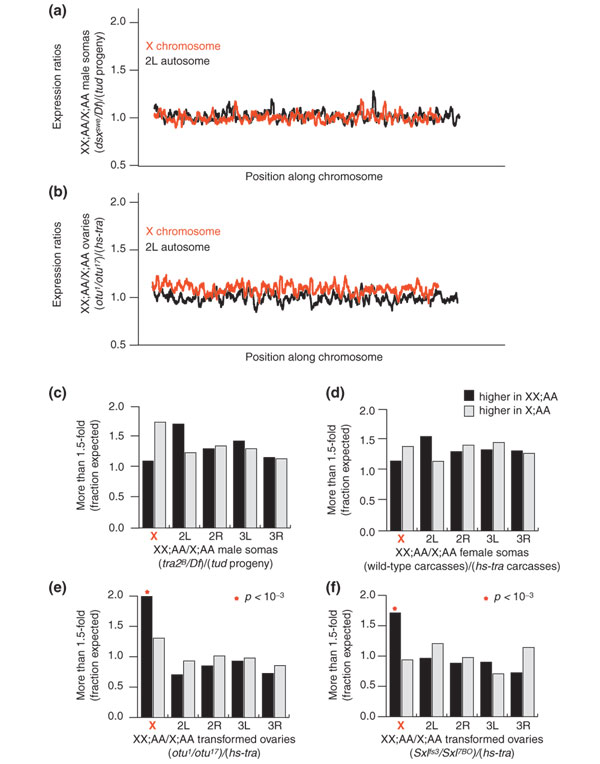
Escaping dosage compensation. **(a,b) **Moving average of expression ratios plotted against position along the X chromosome (red) and autosome 2L (black) from (a) sex-transformed soma and (b) germline. **(c-f) **Histograms of genes showing expression ratios greater than 1.5-fold in either XX;AA (gray) or X;AA (black) transformed soma (c,d) and germline (e,f). Genotypes are indicated. Genes were analyzed by chromosome arm (number of genes >1.5-fold on arm/total on arm)/(number of genes >1.5-fold in genome/total in genome). Χ^2^ tests (*p *> 0.3) indicate that there is no enrichment on the X chromosome for genes expressed more than 1.5-fold in XX;AA soma, but that there is enrichment in gonads (*, *p *< 10^-3^).

Although the overall expression of X-chromosome genes appears to be very tightly dosage compensated, there might be small regions or scattered genes that escape dosage compensation. To look for blocks of the X chromosome that fail to undergo dosage compensation in either the soma or the germline, we plotted a moving average of expression ratios along the length of the X or the similarly sized autosome arm 2L (Figure 8). In the case of the soma, plots of (XX;AA)/(X;AA) expression ratios revealed only noise fluctuating around expression ratios of unity. X-chromosome expression ratios for gonads were slightly skewed towards XX-biased expression, but we detected no overt region of the X chromosome expressed like a *Df*/+ region. These results indicate that no contiguous blocks of genes on the X chromosome escape dosage compensation in the soma or germline.

Scattered genes along the X chromosome that fail to become dosage compensated in either the soma or the germline are more difficult to detect because there are X chromosome and autosomal genes that are differentially expressed between XX;AA and X;AA tissues even following sex transformation of those tissues. If some autosomal genes are expressed at higher levels in XX;AA flies than in X;AA flies, then some X-chromosome genes are expected to do the same. To test for isolated genes that have escaped dosage compensation, we extracted all the genes that were at least 1.5-fold overexpressed in either XX;AA or X;AA flies, normalized them to gene content per arm, and looked for departure from a normal distribution (Figure 8). If there are a significant number of genes on the X chromosome that escape dosage compensation, then there should be more expression outliers mapping to the X chromosome relative to similarly sized autosomal segments. Furthermore, the outliers from the X chromosome should show XX-biased expression if they escape dosage compensation. In the sex-transformed soma, even though there are X-chromosome genes that always show higher expression in XX;AA than X;AA soma, we observed no significant enrichment (Figure 8) of such genes. About twice as many X-chromosome genes as expected, however, show higher expression in XX;AA transformed germline (*p <*10^-3^, by the χ^2 ^test). At face value, we can calculate that approximately 2% of X-chromosome genes escape dosage compensation in the germline. In the soma, sex determination and dosage compensation are linked pathways, however, and this could conceivably be true for the germline [7,22]. We cannot rule out the possibility that reduced X-chromosome expression in X;AA, *hs-tra*/+ ovaries relative to XX;AA *otu *or *Sxl *ovaries is due to a slight defect in germline dosage compensation concurrent with a germline sex transformation.

### Dosage-compensation mechanisms in *C. elegans *and the mouse

Although X-chromosome dosage compensation equilibrates X-chromosome expression between the sexes, the X;AA individuals are the ones to face an unbalanced karyotype. This makes the increased X-chromosome expression in the X;AA male *Drosophila *a logical mechanism of dosage compensation. In contrast, X-chromosome transcription is reduced from both X chromosomes in *C. elegans *hermaphrodites, and one X chromosome is inactivated in mammalian females [15,16]. In both cases, this dosage compensation equilibrates X-chromosome expression between the sexes, but also makes both sexes functionally aneuploid with respect to the autosomes. This conundrum is solved if X chromosomes are generally hypertranscribed and if X-chromosome inactivation or hypotranscription in XX;AA females or hermaphrodites is a mechanism to prevent overexpression of the X chromosome. Otherwise, hypertranscription of the X chromosome in XX;AA individuals would result in functional tetraploidy.

**Figure 9:**
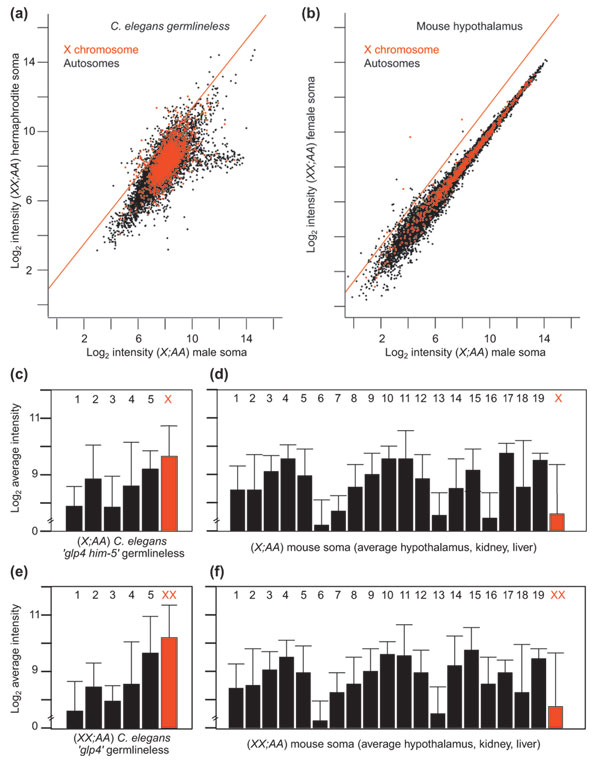
Scatterplots of hybridization intensities from RNA from somatic tissue from XX;AA and X;AA *C. elegans *and mouse. Hybridization intensities of **(a) **germlineless (*glp4*) XX;AA hermaphrodites plotted against a population greatly enriched for germlineless X;AA male *C. elegans *(*glp4 him5*), and **(b) **female mouse hypothalamus tissue plotted against matched tissue from males. X chromosome (red) and autosomal (black) elements as well as the trend line (red) for the twofold expression difference expected in the absence of dosage compensation are shown. **(c-f) **Average hybridization intensities corresponding to genes on the X chromosome (red) and autosomes (black) in (c) X;AA *glp4 him5 C. elegans *male soma and (d) X;AA mouse male soma (average of hypothalamus, kidney and liver) samples, (e) XX;AA *glp4 C. elegans *hermaphrodite soma, and (f) XX;AA mouse female soma samples. The intensities from individual autosomal arms are averaged across all experiments and standard deviations of the means are indicated.

In order to investigate the activity level of the X chromosome in these animals, we reanalyzed previously published array data on gene expression in the soma of hermaphrodite and male *C. elegans *[28] and female and male mice [29]. These studies reported on gene-expression differences between the sexes rather than dosage compensation. From scatterplots of these data there is little difference between the expression of the X chromosome in XX;AA versus X;AA germlineless animals or somatic tissues (Figure 9). Given that both species have somatic dosage-compensation mechanisms that equilibrate expression between the sexes, this is unsurprising.

To determine whether per-copy transcription from the X chromosome is reduced in XX;AA somas, we measured the average hybridization intensity for the X and all autosomes in both X;AA and XX;AA animals (Figure 9). Transcripts from the single X chromosome in X:AA *C. elegans *male soma hybridize as well as those of two-copy autosomes. Indeed, the X chromosomes of both X;AA and XX;AA *C. elegans *appear to be overexpressed relative to autosomes (*p *<< 10^-4^). Similarly, X-chromosome transcripts from X;AA and XX;AA mouse soma (average of hypothalamus, kidney and liver) are detected well within the range of those of the autosomes. Many mouse tissues have been examined, and we find evidence of elevated X-chromosome expression in all cases (data not shown). A study concurrent with this one has extensively cataloged mammalian X-chromosome dosage compensation, and confirms our observation that the single X chromosome in males and the single active X chromosome in females are compensated [30]. These findings suggest that the single X chromosomes of X;AA *C. elegans *and mice are expressed at levels comparable to two autosomes, as we observed in *Drosophila*. Perhaps this is a universal phenomenon.

## Discussion

Polytene salivary gland chromosomes have greatly facilitated the analysis of dosage compensation in *Drosophila *[8], but global analysis of dosage compensation in the other tissues has not been conclusive. A recent reverse transcriptase (RT)-PCR study showed a wide range of states of compensation of X-chromosome genes [31]. Sex-biased expression potentially distorts such dosage-compensation analysis, however. There is extensive sex-biased expression in *Drosophila *[24,32-34] and, furthermore, these genes are not equally distributed between the X chromosome and autosomes [25,35]. Studies directed at subsets of genes have revealed complex relationships between X chromosome and autosome expression. It is, however, difficult to interpret a subtle difference in X-chromosome expression between XX;AA and X;AA individuals if effects of a similar magnitude are seen for autosomal genes [36]. Global analysis of transcription in non-polytene tissues could help resolve the nature of X-chromosome dosage compensation in the understudied tissues that make up the bulk of the fly.

Microarray analysis is ideally suited to the study of dosage compensation. Our results suggest that remarkably tight X-chromosome dosage compensation occurs in a wide range of adult tissues. Most importantly, we also provide the first evidence for germline dosage compensation in any organism. The compensation mechanism in both the soma and germline is the equilibration of X chromosome expression with autosomal expression. This is likely to be due to increased expression from the single X chromosome in X;AA, although decreased expression from all the autosomes could also ensure that X chromosome expression is equilibrated with the autosomes [37].

### MSL-independent germline dosage compensation

Germline X-chromosome dosage compensation has been a black box for decades [22]. As the well-known somatic dosage-compensation mechanisms in the major genetic model organisms clearly do not function in the germline, the idea has gradually emerged that germ cells are dosage-tolerant. This is due not to evidence of absence, but to absence of evidence. Although our results show that dosage compensation in the soma and germline are thematically similar, the mechanisms in the germline must be distinct.

The MSL complexes required for dosage compensation in the soma are dispensable in the germline [10,12]. This might appear to be non-parsimonious, but germline and somatic gene expression also appear to be distinct. The male and female germline express different types of basal transcription machinery, such as male-germline-specific TATA-box-binding associated factors (TAFs), which might require a different chromatin structure [38,39], and germline-restricted factors bind the germline-restricted core promoter of the *ovo *gene [40]. In addition, even though somatic dosage compensation is highly conserved, proteins mediating dosage compensation are not. For example, in some other species of flies the MSL complex associates with all chromosomes, not just the X chromosome [41-43], suggesting that the complex plays no role in dosage compensation in those organisms. In yeast, which lack well differentiated sex chromosomes, one of the main MSL complex components is required for viability [44]. These observations indicate that MSL complexes play markedly different roles in different species. Given the differences in gene expression between the germline and the soma, it is perhaps unsurprising that different proteins might be involved in *Drosophila *germline dosage compensation.

It is also unclear whether the MSL complex accounts for all somatic dosage compensation. For example, XX;AA flies lacking Sex-lethal (Sxl) protein in the soma die, presumably because of a failure to repress MSL complex formation [45]. But mutations in the MSL-encoding genes fail to rescue the lethality of XX;AA flies without Sxl, suggesting that Sxl represses additional dosage-compensation functions [46]. Similarly, in polytene salivary glands, large blocks of the X chromosome do not associate with MSL complexes [47]. We find no evidence to support extensive escape from X-chromosome dosage compensation in somatic tissues. These results are consistent with either selective deployment of MSLs at active genes [47] or MSL-independent dosage compensation at a subset of genes.

Mutations in genes regulating germline dosage compensation would be expected to show karyotype-specific phenotypes. Interestingly, there are at least two genes that appear to be required for the viability of XX;AA but not X;AA germ cells; *ovo *and *stand still *(*stil*) [48,49]. Negative and positive Ovo protein isoforms act at the transcription start site of at least some promoters [5,40,50] and Stil protein decorates active chromatin when overexpressed in the soma [49]. Perhaps one or both of these proteins serve to block the upregulation of the X chromosome that normally occurs in the male germline. This could be analogous to the situation in the *Drosophila *soma, where dosage compensation is actively repressed in XX;AA females [7]. There are also a number of genes that are required for the viability of male germ cells [51]. Some of these genes may encode dosage-compensation functions. If such dosage-compensation genes exist, they might encode chromatin-modifying complexes, as in the *Drosophila *soma. Given that our data show that steady-state transcripts from the X chromosome are compensated, however, there are a host of post-transcriptional mechanisms, such as preferential mRNA stability, that could conceivably mediate germline dosage compensation.

X-chromosome dosage-compensation mechanisms might arise from dosage control mechanisms that are active at many or all genes. Indeed, we have detected transcriptional buffering in our control experiments on the effects of autosome gene dose. We found that the magnitude of the transcriptional effect was less than expected from a simple calculation of gene dose. This inverse effect on transcription relative to gene dose has been known for decades [52-54], and has recently been observed in expression profiling and RT-PCR studies [31,55-57]. It seems likely that many genes have a self-contained dosage-compensation system, albeit an imperfect one. The MSL components have general transcriptional activity in yeast and appear to have been co-opted in *Drosophila *to regulate somatic dosage compensation. Germline dosage compensation might be mediated by other co-opted *cis- *or *trans*-acting components that serve to buffer gene expression.

### Escape from dosage compensation

There are X-chromosome genes, such as *LSP1-α*, that escape dosage compensation in *Drosophila *[58], but it is not known how common this might be. Similarly, in mammals, many X-chromosome genes escape inactivation in females (escape from dosage compensation in X;AA males has not been examined). In theory, this is expected, as not all genes need to be dosage compensated in the course of X-chromosome evolution [59]. Although we can confidently analyze populations of X-chromosome genes, it is quite difficult to determine whether a given gene escapes dosage compensation. Indeed, in the absence of a detailed, gene-by-gene mechanistic study, it might be impossible. There are many autosomal genes that are more highly expressed in XX;AA than X;AA tissues, even after controlling for sex-biased expression. These gene-expression differences are clearly not directly related to X-chromosome dosage compensation. Many of the X-chromosome genes expressed at higher levels in XX;AA than X;AA tissues will be similarly unrelated to dosage compensation *per se*. In studies of escape from dosage compensation in *Drosophila*, *C. elegans*, or mammals where sex-bias is uncontrolled, reports of genes escaping dosage compensation could well be spurious.

On a global level, we find no evidence for escape from dosage compensation in the *Drosophila *soma. In the germline, however, there are clearly more genes with XX-biased expression than expected. There are several different interpretations of this finding. It is possible that escape from dosage compensation is more common in the germline (about 2% of genes). Alternatively, there may be slight under-compensation in the germline. The X chromosomes in both XX;AA and X;AA gonads appear to be overexpressed relative to autosomes, however. It is therefore possible that the slight excess of XX-biased expression is due to failure to fully dampen X-chromosome hypertranscription in XX;AA females.

### Towards a unified model of dosage compensation

**Figure 10:**
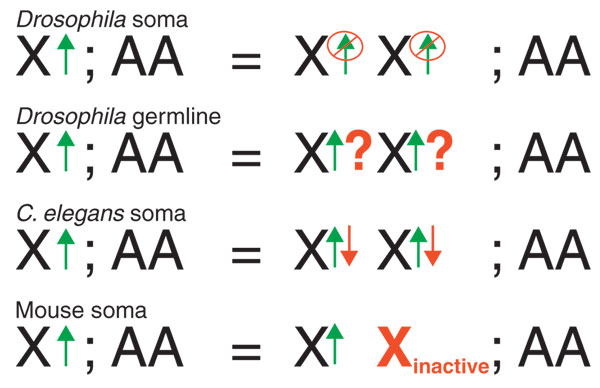
Model for X-chromosome dosage compensation in *Drosophila*, *C. elegans *and mouse. X-chromosome transcription is generally upregulated (green arrow). X;AA tissues thus avoid unbalanced expression of X-chromosome genes. In order to avoid overexpression of X-chromosome genes (red symbols), females either destroy the compensation machinery used in males (*Drosophila *soma), or deploy a counteracting mechanism to reduce expression from both hyperactive X chromosomes (*C. elegans*) or eliminate expression from one hyperactive X chromosome (mouse). The mechanism used in the XX;AA *Drosophila *germline is unknown, but given that X-chromosome expression in the female germline is bi-allelic, an X-inactivation mechanism is unlikely.

Females have two X chromosomes and males have one. It is therefore natural to think of the problem of dosage compensation as a mechanism to equilibrate X chromosome expression between the sexes. But changing the dose of large segments of the genome is a problem for the individual with the aneuploid condition - the males in the case of the major metazoan model systems. Remarkably, an X chromosome is inactivated in female mammals. Equally remarkable, both X chromosomes in *C. elegans *hermaphrodites are downregulated. Why should X chromosome dosage compensation be achieved in these organisms by unbalancing gene expression in both sexes? Our analyses of pre-existing array data suggest that both the male X chromosome and the single active female X chromosome are hypertranscribed or accumulated in mammals. In the course of our study, some of the same data has been reanalyzed independently, and the authors of the other study have reached similar conclusions [30]. These data support a more unified theory of dosage compensation [17-20], whereby X-chromosome expression is increased in all X;AA cells (Figure 10). There may be counteracting forces to increase X-chromosome expression in X;AA males and to keep X-chromosome expression under control in XX;AA females. Indeed, our analysis suggests that in *Drosophila *and *C. elegans*, the X chromosomes of XX;AA and X;AA tissues are overexpressed relative to autosomes. Such overshooting would be expected to be more detrimental to XX;AA individuals and could have given rise to X-chromosome inactivation in the mammalian lineage. Interestingly, the repressive chromatin-associated protein HP1 is enriched on the *Drosophila *X chromosome in males [60]. Perhaps this is to modulate overcompensation.

## Conclusion

Dosage compensation has been under study for a nearly a century. Examination of chromatin structure, rather than direct assay of gene transcription, dominates the recent dosage-compensation literature. Microarray analysis allows us direct access to the transcript accumulation of all X-chromosome genes. The *Drosophila *array data provide the first demonstration in any organism that germ cells dosage compensate. These data also add a significant twist to our understanding of dosage compensation in mammals and *C. elegans*. Microarray analysis will be a vital tool for directly assaying this intriguing whole-chromosome regulation of gene expression.

## Materials and methods

### *Drosophila *strains

Flies were grown at 25°C, aged for 5–6 days, dissected, flash-frozen, and prepared for RNA extraction as described previously [24] (see the Flybase compendium of information on genes, alleles, and phenotypes for additional descriptions [27]). Flies used to test for the effect of chromosomal aberrations were *Dp(2;2)Cam3*/+ and *Df(2L)J-H*/+ that were generated from outcrossing the stocks to *y*^*1*^*w*^*67c *^to remove the balancer chromosomes. In addition to using XX;AA females (*y*^*1*^*w*^*67c*^), we transformed XX;AA flies into males using null mutations of *transformer2 *(*tra2*^*B*^/*Df(2R)trix*) and by using a *dsx *mutation encoding a pre-mRNA that is constitutively spliced into the male-specific form (*dsx*^*swe*^). Flies bearing *dsx*^*swe*^*trans *to a deletion produce Dsx^M ^protein and no Dsx^F ^protein (*dsx*^*swe*^/*Df(3R)dsx*^*M*+*15*^). Similarly, flies null for *tra2 *produce only Dsx^M^. To obtain X;AA flies that are sex transformed from males into females, we crossed female flies bearing female-specific *tra* cDNA transgenes (*w*; *P*{*w*^+ ^*hs-tra*^*83*^}/*CyO *or *Gla*) or *(Df(3L)st*^*j7*^*Ki roe p*^*p*^*P*{*hs-tra*}/*TM3 *or *TM6*) to *B*^*s*^*Y *males. The *hs-tra *transgenes are sufficiently active at 25°C to transform males into phenotypic females. We did not utilize a heat-shock regimen.

In order to study the expression changes resulting from a twofold change in the dose of the X chromosome between the XX;AA and X;AA germlines, we utilized genetic mutations that we show remove most sex-biased germline expression from the analysis. The activation of female rather than male sexual differentiation in the soma with *hs-tra *in X;AA flies results in gonads with vast numbers of poorly differentiated germ cells. Mutations in *Sxl *(*y Sxl*^*fs3*^/*y cm Sxl*^*7BO*^) or *otu *(*ct otu*^1 ^*v*^*24*^/*y w otu*^*17*^) result in a similar germline phenotype. Occasionally, a few ovaries from X;AA *hs-tra *flies and germline-transformed *Sxl *or *otu *flies bear eggs. These ovaries were not included in the samples. Similarly, very small ovaries that are essentially germlineless were not included in any of our samples.

To remove germline expression from the analysis of somatic X-chromosome dosage compensation, we took advantage of the fact that XX;AA flies transformed from females to males usually have no germline, but rarely have a few germ cells showing either oogenic or spermatogenic phenotypes. These germline-atrophic XX;AA females transformed into males were compared with both gonadectomized X;AA males or sham-dissected X;AA males with a genetically ablated germline as a result of the absence of maternal Tud^+ ^(progeny of *tud*^*1*^*bw*^*1*^*sp*^*1*^mothers).

### Arrays

We have used an extensively tested microarray platform designed to detect transcription from 94% of *D. melanogaster *release 1 genes [61]. Unlike most array studies, where the object is to determine which genes are altered between tissues, life stages or treatments, we focus here on non-differential expression. In dozens of homotypic hybridization experiments (where an mRNA sample is split, labeled with either Cy3 or Cy5, mixed, and hybridized to the array) performed as part of this (data not shown) and previous studies, we find that the expression ratio is 1 and that there are very few outliers [24,25,61]. In the most extensive set of such homotypic hybridizations, the 99.5% confidence intervals were always between 1.4 and 1.5-fold [61]. Given that only a handful of genes are expected to show artifactually a greater than 1.5-fold expression difference, we easily had the sensitivity to detect changes in expression owing to a twofold difference in the 2,245 X-chromosome genes represented on the array.

We have further shown that twofold differences in mRNA concentrations can be readily detected by adding known concentrations of mRNA to hybridization mixes in 'spike-in' experiments [61]. Analysis of spike-in control data (a twofold change in input results in a twofold difference in expression ratios) and a comparison of sex-biased expression determined by FlyGEM and northern blotting reveal no evidence of compression of expression ratios in our data [24,61].

### Sample preparation and labeling

We used 96 biological samples in this study. Careful sample preparation ensures minimum adjustment in subsequent data handling. Dissections, flash freezing and RNA extractions were performed as described [24,61]. Briefly, total RNA was extracted using Trizol (Life Technologies, Carlsbad, USA), followed by mRNA isolation using an Oligotex poly(A) extraction kit (Qiagen, Valencia, USA). RNA concentration was determined using RiboGreen dye (Molecular Probes, Oak Ridge, USA) in a fluorescent assay using a Luminescence Spectrometer LS-50B (PerkinElmer, Fremont, USA) fitted with 485 nm (excitation) and 520 nm (emission) filters. RNA quality was determined by capillary electrophoresis on a Bioanalyzer 2100 (Agilent, Palo Alto, USA), using the 6000 Nano Assay kit (Agilent) according to the manufacturer's instructions.

Samples were labeled with Cy3- or Cy5-labeled random nonamers (Trilink Biosciences, San Diego, USA). To ensure that each of the nonamer sets was in fact equally 'random', we ordered a single batch of random oligonucleotides that was split into half at the penultimate synthetic step with Cy3 or Cy5 nucleotides added at the end. To verify performance of these oligonucleotides, we performed an experiment in which RNA was labeled using both Cy3 and Cy5 nonamers in the same tube, followed by hybridization, scanning and analysis. There was no dye bias evident. Hybridizations of samples to the microarrays were performed at 60°C, followed by washes exactly as described [24,61].

### Scanning

Arrays were scanned using an Axon GenePix 3000A fluorescence reader (Molecular Devices Corporation, Union City, USA) in which the photon multiplier tube (PMT) settings were first adjusted using calibration slides. GenePix v.4.1 image acquisition software (Molecular Devices Corporation) was used to extract signal for each target element. Calibration slides were Ultra GAPS (Corning, Acton, USA) spotted with 10^-3 ^to 10^-6 ^dilutions of a 0.33 nM each stock of Cy3 and Cy5 dyes (Amersham Biosciences, Pittsburgh, USA) using GMS 417 Arrayer (Affymetrix, Santa Clara, USA). The PMT voltages, in the linear range, were balanced using these slides. With each new batch of labeling and hybridization, we included a homotypic hybridization [61], in which the same RNA preparation from *y*^1 ^*w*^*67c *^whole males was split into two tubes, labeled with Cy3 or Cy5 nonamers, mixed and hybridized. Slight adjustments of PMT voltages were sometimes made on these control slides. No elements were saturated. Once PMT settings were determined, all arrays in the same hybridization batch were scanned at the same voltage settings. Hybridization and labeling quality was determined by confirming that the channels were close to global balance in the absence of PMT adjustment (no 'on-the-fly' adjustments were made). No arrays were discarded because of failed labeling reactions in one of the two channels. In addition, all elements were printed in duplicate. Regression analysis of plots of duplicate elements was also used as a quality-control step for gradients and severe speckling. No arrays were discarded following duplicate element evaluation. Finally, we determined the fraction of elements that returned signal above on-spot background (see Data handling). Three arrays were discarded from the study because less than 50% of elements exhibited above-background signal.

### Loop design for microarray pairings

Samples from all flies and tissues were prepared (Table 1) and hybridized using a loop design [62]. This design (see Additional data file 2) ensured first, that the highest-quality direct hybridizations were predominantly between matched XX;AA and X;AA tissues and second, that any sample could be compared with every other sample included in the study, after normalization across all samples. Most of the relevant samples were compared directly, but all samples are connected in the design for indirect comparisons. In most direct comparisons we have included both technical labeling replicates (dye-flips) and biological replicates. Although we used a series of pair-wise comparisons of XX;AA and X;AA samples in this study, the flexible design will allow easy future use to address other questions about sex-biased expression.

### Data handling

*Drosophila *data were processed using the Bioconductor [63] package limma (Linear Models for Microarray Analysis) v. 1.7.6, to adjust for effects that arise from variations in microarray technology rather than biological differences. Because of the care taken in using identical RNA concentrations in labeling reactions and scanner settings, data handling resulted in minimal adjustments to the raw data. We did not average duplicate array elements as this would reduce statistical power in later steps. At the individual array level, the raw intensity data were normalized using print-tip loess (locally weighted scatter plot smooth) [64]. This corrects for tip biases and for minor hybridization gradients. This was followed by one of three different background corrections. For background elimination, intensities less than the average intensities of the barcode elements (designed against DNA corresponding to an intron that were included in printing plates for tracking purposes, but are also quite useful for background correction [61]) were classified as background and excluded from calculations. For on-spot background correction, the average intensity of the barcoding elements in the array were subtracted from the spot intensity for every array element. For the no-correction option, we used quantile normalization to compare across arrays [64]. Experiments with the *Df*/+ and *Dp*/+ flies confirm that on-spot background correction maximizes expression differences within aneuploid segments, but the effect of the copy number was evident with both background elimination and no background correction (see Additional data file 1).

Array data from *C. elegans *and mouse were extracted directly from the Gene Expression Omnibus (GEO) website [65]. We performed no additional normalization or background correction. The *C. elegans *experiments were two-channel hybridizations on spotted arrays [28]. Test samples were hybridized with a reference sample. The reference channels cancel in our comparison of XX;AA vs X;AA gene expression. The mouse experiments were performed on Affymetrix arrays [29].

### Data sources

All array data are available at GEO. An extensive platform description of FlyGEM is available at GEO under accession number GPL20 [61]. Data are available under GEO accessions GSM37438-GSM37451, GSM2464-2467, GSM16582-GSM16583, GSM16570-GSM16581, and GSM77749-GSM77752. Expression data from mouse hypothalamus, kidney and liver were obtained from GEO accession number GSE1148 [29]. *C. elegans *data were from GEO accession number GSE715 and GSE724 [28].

### Exploratory data visualization

Data were clustered and visualized using Cluster 3.0/Tree-View 1.6 [66]. A self-organizing map (SOM) was calculated to organize normalized hybridization intensities (on-spot background subtraction followed by quantile normalization across arrays) of array elements at 100,000 iterations, followed by k-means clustering of genes with 10 nodes using the correlation (uncentered) similarity metric. Genes, but not experiments, were clustered. We averaged duplicate elements for this analysis to avoid crashing the application.

For scatterplots and moving average plots, we used data processed by background elimination followed by quantile normalization across all experiments. We used on-spot background corrected data for histograms of *Drosophila *expression data. Chi-squared tests were performed in Excel (Microsoft, Redmond, USA).

Array elements encoding *Drosophila *ribosomal subunits were identified using the Gene Ontology identifier 'structural constituent of ribosomes' as assigned in GEO accession GPL20. The *de facto *housekeeping elements were identified as follows. Following background elimination and quantile normalization across all experiments, we selected the elements with hybridization intensities greater than two standard deviations above the median intensity of all array elements, from all experiments. From the above, we selected the *de facto *housekeeping elements as those elements with standard deviations for the expression ratios as ± 0.1. Regressions for the X-encoded and the autosome-encoded elements were calculated separately in Bioconductor. Slopes and intercepts were compared at the 95% confidence limit.

Moving averages of expression ratios for every 40 consecutive elements (two elements per predicted transcript) were plotted against the corresponding gene positions on chromosome arm 2L or X. The predicted cytological break points [27] were used to define the boundaries of aneuploid segments on chromosome arm 2L, in the *Dp*/+ versus *Df*/+ experiments. Moving averages were calculated in Bioconductor.

### Analysis of distributions

The distribution statistics cited in the text were obtained using data processed with print-tip loess and on-spot background correction on individual arrays, and quantile normalization between arrays. We can unequivocally show that a modest 1.5-fold difference in gene dose has a detectable effect on gene expression using precisely the same data-handling methods that we apply to the study of the X. In addition, the data-handling method used is the least favorable method *vis-a-vis *the conclusions we report. Differences in gene expression in autosomal aneuploid segments were always significantly greater than those observed between the X chromosomes from XX;AA and X;AA samples.

We performed a one-way analyses of variance (ANOVA) followed by the Tukey HSD method for protection in multiple comparisons using Matlab (The MathWorks, Natick, USA). All cells were filled ten times. One-way ANOVA was used to compare three different sets of expression ratios or hybridization intensities. The single blocking factor for one-way ANOVA was gene dose. We used bootstrap sampling of pooled array data to fill each of the three ANOVA cells of the one-way design. For ratiometric data, those cells were (*Dp*/+)/(*+*/+), AA/AA, and XX/X (see Additional data files 3, Tables 1-3). To fill the (*Dp*/+)/(*+*/+) cell, we pooled expression ratios associated with a 1.5-fold difference in gene dose from the replicate samples from whole males, whole females and ovaries, and sampled 600 ratios. There are fewer genes in the *Dp *region than on all autosomes or on the X chromosome. Therefore, the stability of this inference was verified by filling this cell with 200 ratios from each of the tissue types separately (data not shown). To fill the AA/AA cell we pooled all array data reported in this article where the gene dose was 1 and sampled 600 ratios. To fill the XX/X cells we sampled 600 X-chromosome ratios for each of three different gonad comparisons separately. Those are (*otu*^1^/*otu*^17^)/(*hs-tra*/+), (*Sxl*^*fs*3^/*Sxl*^7*BO*^)/(*hs-tra*/+) and ovary/testis (see Additional data file 3, Tables 1-3). For analysis of *Drosophila *intensity data, the cells were *Df*/+, AA, and X (see Additional data files 3, Tables 4,5). As with ratiometric data, intensity data were bootstrap sampled 600 times and the procedure was repeated ten times. Stability for the *Df*/+ cell was also verified. The inferences were similar in all ten samplings of 200 intensity values from each tissue type (data not shown). For *C. elegans *and mouse data the cells were AA and X (see Additional data file 3, Table 6). These cells were filled as for *Drosophila*. For mouse, we ran ANOVA on pooled intensities from liver, kidney and hypothalamus. We also analyzed each tissue separately. The Tukey HSD procedure was performed in Matlab and generated a confidence interval for each pair of comparisons that has a family-wise error rate of, at most, α = 0.05 (see Additional data file 3).

For the *Drosophila *data, we applied a bootstrapped Hodges-Lehmann (HL) estimate of median differences to determine if X-chromosome expression was more similar to autosomal expression or to expression from autosomal aberrations (see Additional data files 4,5). Additional data file 6 shows a cartoon depicting bootstrap HL estimates of median differences followed by the Kolmogorov-Smirnov (KS) test. HL estimates and KS tests were performed in Bioconductor. For ratiometric data (see Additional data file 4), the HL estimate was calculated for every drawing of 600 bootstrap samples from the (*Dp*/+)/(+/+) and AA/AA distributions to generate a new distribution of differences. Next, the same bootstrap sampling was used for the XX/X and AA/AA distribution to generate a second distribution of differences. Intensity data were treated similarly (see Additional data file 5). The two distributions of differences were then compared using the KS test. The differences in the medians and associated *D *and *p*-values for the KS tests then permitted inference concerning the proximity of expression ratios or intensities. In practical terms, a significant *p*-value allows us to accept (not reject) the assumption that expression ratios AA/AA are closer to XX/X than to *Dp*/+. This procedure of bootstrap sampling, HL estimation and KS evaluation was repeated ten times to check the stability and robustness of the inferences. In all cases, replicate evaluations resulted in the same inference.

## Additional data files

The following files are available. Additional data file 1 is a figure showing the effect of different data handling techniques on differential expression resulting from altered gene dose on the autosomes. Additional data file 2 is a figure showing the experimental design in detail. Additional data files 3,4and 5 are tables showing the results of statistical analysis of gene expression ratios and absolute intensities for whole chromosome arms and for autosomal aneuploid segments: Additional data file 3 shows one-way ANOVA comparisons and Additional data file 4 shows estimation of Hodges-Lehmann (HL) median differences between expression ratios in various experiments and Additional data file 5 shows estimation of Hodges-Lehmann (HL) median differences between signal intensities. Additional data file 6 is a figure showing a simple picture depicting bootstrap HL estimates of median differences followed by the Kolmogorov-Smirnov (KS) test.

## Supplementary Material

Additional data file 1 A figure showing the effect of different data handling techniques on differential expression resulting from altered gene dose on the autosomes Click here for additional data file

Additional data file 2 A figure showing the experimental design in detail Click here for additional data file

Additional data file 3 One-way ANOVA comparisons Click here for additional data file

Additional data file 4 Estimation of Hodges-Lehmann (HL) median differences between expression ratios in various experiments Click here for additional data file

Additional data file 5 Estimation of Hodges-Lehmann (HL) median differences between signal intensities in various experiments Click here for additional data file

Additional data file 6 A figure showing a simple picture depicting bootstrap HL estimates of median differences followed by the Kolmogorov-Smirnov (KS) test Click here for additional data file

## Figures and Tables

**Table 1:** Samples used for measuring X chromosome dosage compensation

Karyotype	Somatic phenotype	Germline phenotype	Sample	Genotype	*N*
XX;AA	Female	Oogenic	Wild type (whole fly)	*y*^*1*^*w*^*67c*^/+; *Dp(2;2)Cam3*/+	2
XX;AA	Female	Oogenic	Wild type (whole fly)	*y*^*1*^*w*^*67c*^/+; *Df(2L)J-H*/+	2
XX;AA	Female	Oogenic	Wild type (ovary)	*y*^*1*^*w*^*67c*^/+; *Dp(2;2)Cam3*/+	2
XX;AA	Female	Oogenic	Wild type (ovary)	*y*^*1*^*w*^*67c*^/+; *Df(2L)J-H*/+	2
X;AA	Male	Spermatogenic	Wild type (whole fly)	*y*^*1*^*w*^*67c*^/*Y; Dp(2;2)Cam3*/+	2
X;AA	Male	Spermatogenic	Wild type (whole fly)	*y*^*1*^*w*^*67c*^/*Y; Df(2L)J-H*/+	2
XX;AA	Female	Oogenic	Wild type (whole fly)	*y*^*1*^*w*^*67* *c*^	5
XX;AA	Female	Ovaries removed	Wild type (carcass)	*y*^*1*^*w*^*67* *c*^	8
XX;AA	Female	Oogenic	Wild type (ovary)	*y*^*1*^*w*^*67* *c*^	12
XX;AA	Female	Germlineless	*tud*^*1*^progeny (whole fly)	*tud*^*1*^*bw*^*1*^*sp*^*1*^	5
XX;AA	Transformed to male	Atrophic	*dsx*^*swe*^/*Df *(whole fly)	*dsx*^*swe*^/*Df(3R)dsx*^*M*+*15*^	6
XX;AA	Transformed to male	Atrophic	*tra2*^*B*^/*Df *(whole fly)	*tra2*^*B*^/*Df(2R)trix*	8
XX;AA	Female	Non-differentiated 'ovarian tumor'	*otu*^*17*^/*otu*^*1*^(ovary)	*ct otu*^*1*^*v*^*24*^/*y w otu*^17^	8
XX;AA	Female	Non-differentiated 'ovarian tumor'	*Sxl*^*fs3*^/*Df *(ovary)	*y Sxl*^*fs3*^/*y cm Sxl*^*7BO*^	9
X;AA	Male	Spermatogenic	Wild type (whole fly)	*y*^*1*^*w*^*67* *c*^	9
X;AA	Male	Testes removed	Wild type (carcass)	*y*^*1*^*w*^*67* *c*^	7
X;AA	Male	Germlineless	*tud*^*1*^progeny (whole fly)	*tud*^*1*^*bw*^*1*^*sp*^*1*^	5
X;AA	Transformed to female	Ovaries removed	*hs-tra *(carcass)	*y*^*1*^*w*^*67c*^/*Y; Df(3L)st*^*j7*^*Ki roe p*^*p *^*P*{*hs-tra*}/+)	2
X;AA	Transformed to female	Ovaries removed	*hs-tra *(carcass)	*w/B*^*s*^*Y; P*{*w*^+ ^*hs-tra*^*83*^}/+	1
X;AA	Male	Spermatogenic	Wild type (testis)	*y*^*1*^*w*^*67* *c*^	10
X;AA	Female	Non-differentiated 'ovarian tumor'	*hs-tra *(ovary)	*y*^*1*^*w*^*67c*^/*Y; Df(3L)st*^*j7*^*Ki roe p*^*p *^*P*{hs-tra}/+)	4
X;AA	Female	Non-differentiated 'ovarian tumor'	*hs-tra *(ovary)	*w/B*^*s*^*Y; P*{*w*^+ ^*hs-tra*^83^}/+	3
