# Supplementary material for: Global analysis of X-chromosome dosage compensation
Source: J Biol. 2006 Feb 16;5(1):3. doi: 10.1186/jbiol30 (PMC1414069; doi:10.1186/jbiol30)
Supplement: Additional data file 1 — A figure showing the effect of different data handling techniques on differential expression resulting from altered gene dose on the autosomes [file jbiol30-s1.pdf]

Additional data file 1

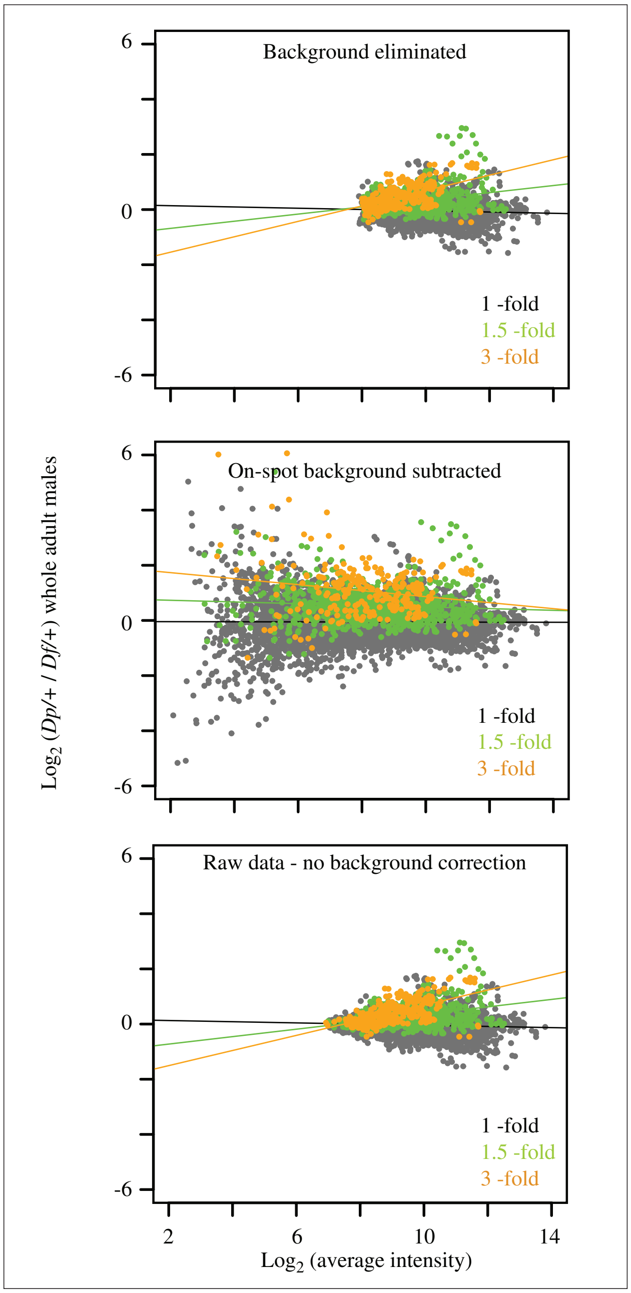

Figure
Effect of background correction method on fold changes in expression ratios. Data were normalized by print-tip loess within each array, and background corrected by one of the following methods: (a) background elimination, (b) on-spot background substraction, (c) no background correction. Background correction impacts the absoluate values of expression ratios. The on-spot background correction which maximizes differences in the data is used in our analyses.
