# Supplementary figures and images for: Global analysis of X-chromosome dosage compensation
Source: J Biol. 2006 Feb 16;5(1):3. doi: 10.1186/jbiol30 (PMC1414069; doi:10.1186/jbiol30)

Additional data file 2

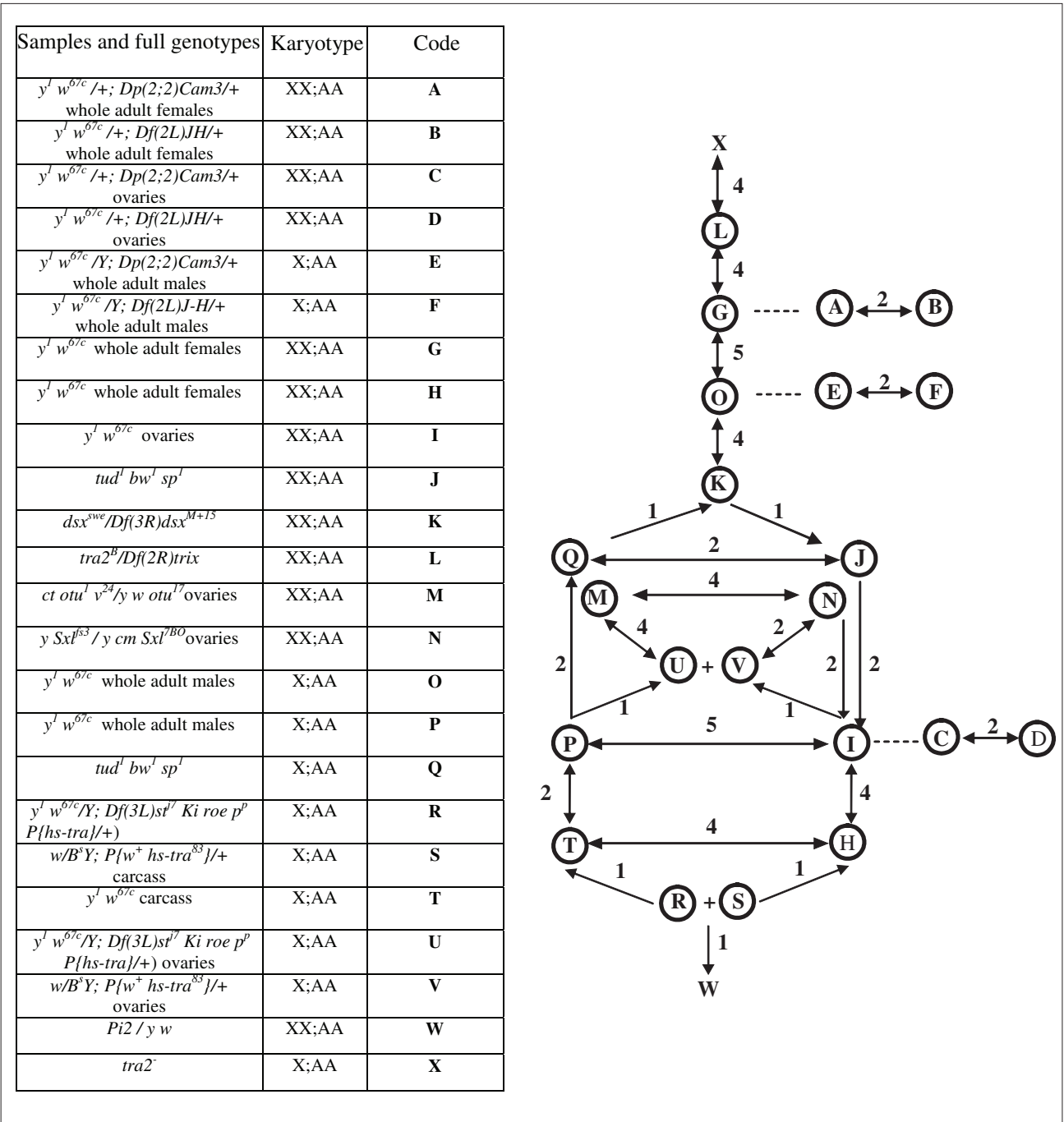

Figure  
The experimental design.

Supplement: Additional data file 2 — A figure showing the experimental design in detail [file jbiol30-s2.pdf]
