# Supplementary material for: Global analysis of X-chromosome dosage compensation
Source: J Biol. 2006 Feb 16;5(1):3. doi: 10.1186/jbiol30 (PMC1414069; doi:10.1186/jbiol30)
Supplement: Additional data file 3 — One-way ANOVA comparisons [file jbiol30-s3.pdf]

Additional data file 3 - Table 1

One-way ANOVA comparing expression ratios of XX;AA (*otu*^{1}/*otu*^{17}) vs. X;AA (*hs-tra*/+) transformed ovaries.

ANOVA cell 1	ANOVA cell 2	ANOVA cell 3
pooled (*Dp*/+)/(+/+)	pooled AA/AA	XX/X
1.5-fold gene dose	1-fold gene dose	2-fold gene dose

Pair-wise comparisons	Mean expression ratio difference and 95% CI
(*Dp*/+)/(+/+) vs. AA/AA	0.4530 [0.4217, 0.4842]
(*Dp*/+)/(+/+) vs. XX/X	0.2808 [0.2496, 0.3121]
XX/X vs. AA/AA	0.1722 [0.1409, 0.2034]

(CI) Tukey HSD procedure confidence interval.

Additional data file 3 - Table 2

One-way ANOVA comparing expression ratios of XX;AA (*Sxl*^{fs3}/*Sxl*^{7BO}) vs. X;AA (*hs-tra*/+) transformed ovaries.

ANOVA cell 1	ANOVA cell 2	ANOVA cell 3
pooled (*Dp*/+)/(+/+)	pooled AA/AA	XX/X
1.5-fold gene dose	1-fold gene dose	2-fold gene dose

Pair-wise comparisons	Mean expression ratio difference and 95% CI
(*Dp*/+)/(+/+) vs. AA/AA	0.4290 [0.3961, 0.4619]
(*Dp*/+)/(+/+) vs. XX/X	0.3069 [0.2740, 0.3398]
XX/X vs. AA/AA	0.1221 [0.0892, 0.1551]

(CI) Tukey HSD procedure confidence interval.

Additional data file 3 - Table 3

One-way ANOVA comparing expression ratios of XX;AA (wildtype) ovaries *vs.* X;AA (wildtype) testes.

ANOVA cell 1	ANOVA cell 2	ANOVA cell 3
pooled (*Dp*/+)/(+/+)	pooled AA/AA	XX/X
1.5-fold gene dose	1-fold gene dose	2-fold gene dose

Pair-wise comparisons	Mean expression ratio difference and 95% CI
(*Dp*/+)/(+/+) *vs.* AA/AA	0.4530 [0.4073, 0.4987]
(*Dp*/+)/(+/+) *vs.* XX/X	0.0319 [-0.0138, 0.0776]
XX/X *vs.* AA/AA	0.4211 [0.3754, 0.4668]

(CI) Tukey HSD procedure confidence interval.

Additional data file 3 - Table 4

One-way ANOVA for intensities from transformed X;AA *hs-tra* ovaries.

ANOVA cell 1	ANOVA cell 2	ANOVA cell 3
Df/+ (1-copy)	Autosomal (2-copy)	X (1-copy)

Pair-wise comparison	Mean intensity difference and 95% CI
Df/+ *vs.* AA	-0.7058 [-0.8793, -0.5324]
Df/+ *vs.* X	-0.8740 [-1.0475, -0.7006]
X *vs.* AA	0.1682 [0.0053, 0.3416]

(CI) Tukey HSD procedure confidence interval.

Additional data file 3 - Table 5

One-way ANOVA for intensities from X;AA wildtype testes

ANOVA cell 1	ANOVA cell 2	ANOVA cell 3
Df/+ (1-copy)	Autosomal (2-copy)	X (1-copy)

Pair-wise comparison	Mean intensity difference and 95% CI
Df/+ *vs.* AA	-0.6785 [-0.8558, -0.5012]
Df/+ *vs.* X	-0.7449 [-0.9222, -0.5676]
X *vs.* AA	-0.0664 [-0.2437, 0.1109]

(CI) Tukey HSD procedure confidence interval.

Additional data file 3 - Table 6

One-way ANOVA comparing hybridization intensities from *C. elegans* or mouse soma

ANOVA cell 1	ANOVA cell 2
Autosomal (2-copy)	X chromosome (1-copy)

Tissue	Pair-wise comparison	Mean intensity difference and 95% CI
C. elegans soma	X vs. AA	0.3933 [0.3547, 0.4320]
Mouse soma	X vs. AA	-0.1704 [-0.3128, 0.0280]
	Chromosome 6 *vs.* AA	-0.2919 [-0.4029, -0.1809]

(CI) Tukey HSD procedure confidence interval.
