# Supplementary material for: Global analysis of X-chromosome dosage compensation
Source: J Biol. 2006 Feb 16;5(1):3. doi: 10.1186/jbiol30 (PMC1414069; doi:10.1186/jbiol30)
Supplement: Additional data file 4 — Estimation of Hodges-Lehmann (HL) median differences between expression ratios in various experiments [file jbiol30-s4.pdf]

Additional data file 4 - Table 1

HL estimation of median differences between expression ratios for XX;AA (*otu^{1}/otu^{17}*) vs. X;AA (*hs-tra*/+) ovaries.

Distribution of ratios (group 1)	Distribution of ratios (group 2)	Median of distribution differences
(*Dp*/+)/(+/+)	AA/AA	0.3689
XX/X	AA/AA	0.1739

D (KS statistic) = 1 ($p << 10^{-4}$) for the two distributions of differences.

Additional data file 4 - Table 2

HL estimation of median differences between expression ratios for XX;AA (*Sxl*^{fs3}/*Sxl*^{7BO}) vs. X;AA (*hs-tra*/+) ovaries

Distribution of ratios (group 1)	Distribution of ratios (group 2)	Median of distribution differences
(*Dp*/+)/(+/+)	AA/AA	0.3689
XX/X	AA/AA	0.1553

D (KS statistic) = 1 (p << 10^{-4}) for the two distributions of differences

Additional data file 4 - Table 3

HL estimation of median differences between expression ratios for XX;AA (wild type) ovaries vs. X;AA (wild type) testes

Distribution of ratios (group 1)	Distribution of ratios (group 2)	Median of distribution differences
(*Dp*/+)/(+/+)	AA/AA	0.3689
XX/X	AA/AA	0.5300

D (KS statistic) = 1 ($p << 10^{-4}$) for the two distributions of differences
