# Supplementary material for: Global analysis of X-chromosome dosage compensation
Source: J Biol. 2006 Feb 16;5(1):3. doi: 10.1186/jbiol30 (PMC1414069; doi:10.1186/jbiol30)
Supplement: Additional data file 5 — Estimation of Hodges-Lehmann (HL) median differences between signal intensities in various experiments [file jbiol30-s5.pdf]

Additional data file 5 - Table 1

HL estimation of median differences for intensities from X;AA *hs-tra* ovaries.

Distribution of intensities (group 1)	Distribution of intensities (group 2)	Median of distribution differences
Df/+	AA	-0.5581
X	AA	0.1631

D (KS statistic) = 1 ($p << 10^{-4}$) for the two distributions of differences.

Additional data file 5 - Table 2

HL estimation of median differences for intensities from X;AA wild type testes.

Distribution of intensities (group 1)	Distribution of intensities (group 2)	Median of distribution differences
Df/+	AA	-0.558
X	AA	0.0153

D (KS statistic) = 1 ($p << 10^{-4}$) for the two distributions of differences
