# Supplementary material for: Global analysis of X-chromosome dosage compensation
Source: J Biol. 2006 Feb 16;5(1):3. doi: 10.1186/jbiol30 (PMC1414069; doi:10.1186/jbiol30)
Supplement: Additional data file 6 — A figure showing a simple picture depicting bootstrap HL estimates of median differences followed by the Kolmogorov-Smirnov (KS) test [file jbiol30-s6.pdf]

Additional data file 6

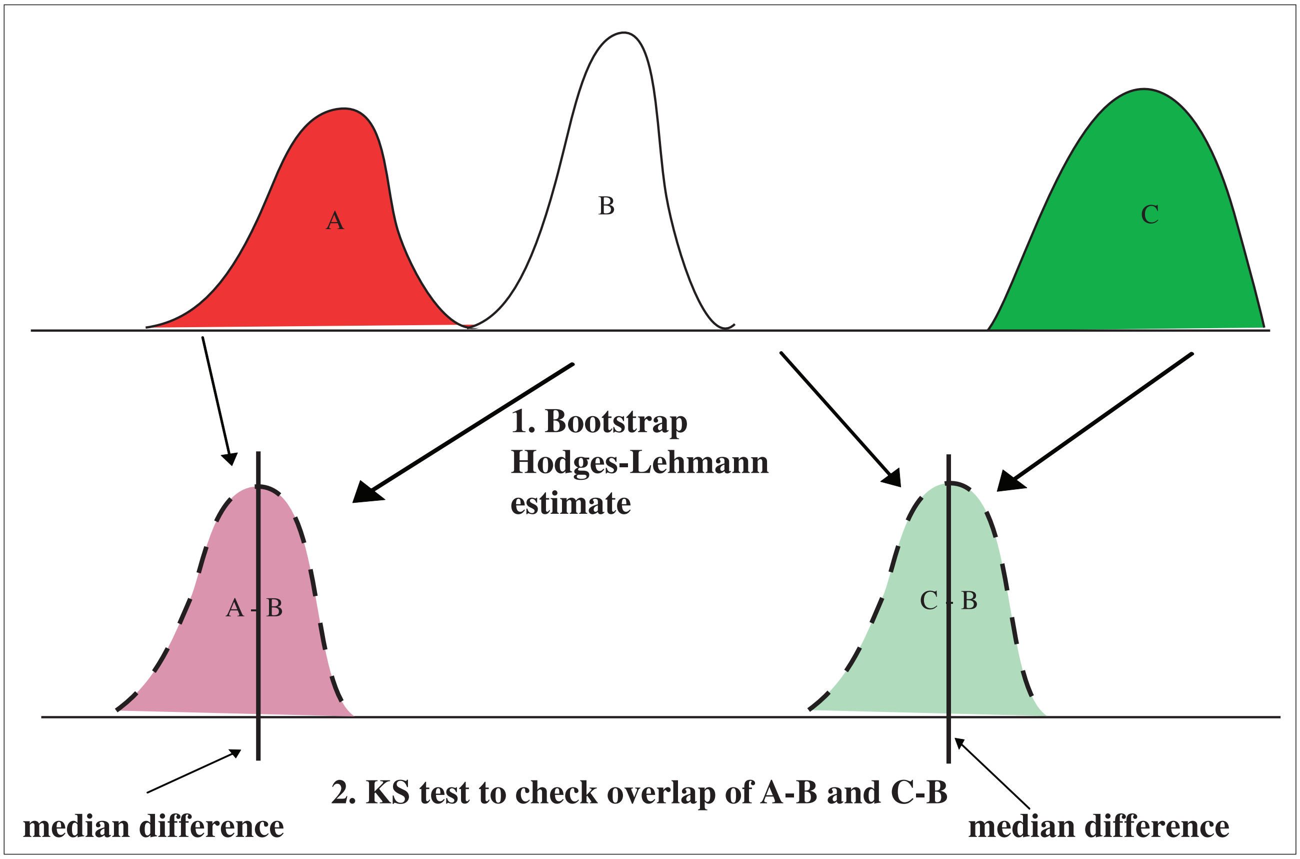

Figure
A simple picture of the Hodges-Lehmann estimate of median differences. Expression ratios (or hybridization intensities) are selected by bootstrap from each distribution. The differences between A and B and B and C are calculated and their distributions are shown. The median differences are indicated, which tell us whether B is closer to A or to C. Their distributions of differences A-B and C-B are compared by KS test, to check for overlap. The *D* statistics of 1 and p-value << 10^{-4} indicate no overlap between A-B and C-B, implying that the B distribution is much closer in median difference to A than it is to C.
